# Risk Prediction Model of Early-Onset Preeclampsia Based on Risk Factors and Routine Laboratory Indicators

**DOI:** 10.3390/life13081648

**Published:** 2023-07-28

**Authors:** Yuting Xue, Nan Yang, Xunke Gu, Yongqing Wang, Hua Zhang, Keke Jia

**Affiliations:** 1 Department of Laboratory Medicine, Peking University Third Hospital, Beijing 100191, China; 2011210372@bjmu.edu.cn; 2 Department of Blood Transfusion, Peking University Third Hospital, Beijing 100191, China; yangnan@bjmu.edu.cn; 3 Department of Obstetrics and Gynecology, Peking University Third Hospital, Beijing 100191, China; guxunke@163.com; 4 Research Center of Clinical Epidemiology, Peking University Third Hospital, Beijing 100191, China; zhanghua824@163.com

**Keywords:** early-onset preeclampsia, risk factors, routine laboratory indicators, risk prediction model, machine learning

## Abstract

**Background:** Globally, 10–15% of maternal deaths are statistically attributable to preeclampsia. Compared with late-onset PE, the severity of early-onset PE remains more harmful with higher morbidity and mortality. **Objective:** To establish an early-onset preeclampsia prediction model by clinical characteristics, risk factors and routine laboratory indicators were investigated from pregnant women at 6 to 10 gestational weeks. **Methods:** The clinical characteristics, risk factors, and 38 routine laboratory indicators (6–10 weeks of gestation) including blood lipids, liver and kidney function, coagulation, blood count, and other indicators of 91 early-onset preeclampsia patients and 709 normal controls without early-onset preeclampsia from January 2010 to May 2021 in Peking University Third Hospital (PUTH) were retrospectively analyzed. A logistic regression, decision tree model, and support vector machine (SVM) model were applied for establishing prediction models, respectively. ROC curves were drawn; area under curve (AUC^ROC^), sensitivity, and specificity were calculated and compared. **Results:** There were statistically significant differences in the rates of diabetes, antiphospholipid syndrome (APS), kidney disease, obstructive sleep apnea (OSAHS), primipara, history of preeclampsia, and assisted reproductive technology (ART) (*p* < 0.05). Among the 38 routine laboratory indicators, there were no significant differences in the levels of PLT/LYM, NEU/LYM, TT, D-Dimer, FDP, TBA, ALP, TP, ALB, GLB, UREA, Cr, P, Cystatin C, HDL-C, Apo-A_1_, and Lp(a) between the two groups (*p* > 0.05). The levels of the rest indicators were all statistically different between the two groups (*p* < 0.05). If only 12 risk factors of PE were analyzed with the logistic regression, decision tree model, and support vector machine (SVM), and the AUC^ROC^ were 0.78, 0.74, and 0.66, respectively, while 12 risk factors of PE and 38 routine laboratory indicators were analyzed with the logistic regression, decision tree model, and support vector machine (SVM), and the AUC^ROC^ were 0.86, 0.77, and 0.93, respectively. **Conclusions:** The efficacy of clinical risk factors alone in predicting early-onset preeclampsia is not high while the efficacy increased significantly when PE risk factors combined with routine laboratory indicators. The SVM model was better than logistic regression model and decision tree model in early prediction of early-onset preeclampsia incidence.

## 1. Introduction

Globally, 10–15% of all maternal deaths can be attributed to preeclampsia or eclampsia, a placentally derived disease of pregnancy [1,2]. Maternal complications associated with preeclampsia include placental abruption, acute kidney disease, pulmonary edema, and heart failure. In severe cases, preeclampsia leads to eclamptic seizures and life-threatening hemolysis, elevated liver enzymes, and low platelet count (HELLP) syndrome [3]. Moreover, fetal complications related to preeclampsia include impaired fetal growth, neonatal respiratory distress syndrome, and stillbirth. Preeclampsia can be classified as early-onset preeclampsia, which develops before 34 weeks’ gestation, and the more common late-onset preeclampsia, which develops at or after 34 weeks’ gestation [4]. Compared with late-onset PE, the severity of early-onset PE remains more harmful with higher morbidity and mortality [5].

Despite the serious clinical consequences, there is no effective preventive measure for preeclampsia currently. Timely identification and management of preeclampsia can significantly improve maternal and perinatal outcomes [6]. Therefore, risk prediction of preeclampsia and preeclampsia-related disorders has received considerable attention over the past two decades. A practical prediction model would allow for increased surveillance of at-risk patients and reduce the surveillance of patients who are less likely to develop preeclampsia, which makes medical resources fully and reasonably allocated and utilized. Although previous studies have analyzed clinical features and evaluated biomarkers for effective prediction, few have demonstrated clinically sufficient properties [7,8,9,10,11].

Machine learning (ML) techniques provide the possibility to infer important connections between items from different data sets that would otherwise be difficult to correlate [12,13]. Due to the vast amount and complexity of medical information, ML is considered a promising method for diagnosing diseases or predicting clinical outcomes. Multiple ML techniques have been used in clinical settings and shown to be more accurate than traditional methods in predicting disease [14].

This study was aimed to develop ML models to predict early-onset preeclampsia by using risk factors and routine laboratory indicators and to compare the performance of different models.

## 2. Materials and Methods 

### 2.1. Study Population

Pregnant women with non-singleton, miscarriage or fetal death, intrauterine chromosomal disorders or fetal malformations, and missing laboratory data were excluded. Preeclampsia (PE) is defined as systolic blood pressure at ≥140 mm Hg and/or diastolic blood pressure at ≥90 mm Hg on at least two occasions measured 4 h apart in previously normotensive women and is accompanied by one or more of the following new-onset conditions at or after 20 weeks of gestation: 1. Proteinuria (i.e., ≥30 mg/mol protein:creatinine ratio; ≥300 mg/24 h; or ≥2 + dipstick); 2. Evidence of other maternal organ dysfunction, including acute kidney injury (creatinine ≥90 μmol/L; 1 mg/dL); liver involvement (elevated transaminases, e.g., alanine aminotransferase or aspartate aminotransferase >40 IU/L) with or without right upper quadrant or epigastric abdominal pain; neurological complications (e.g., eclampsia, altered mental status, blindness, stroke, clonus, severe headaches, and persistent visual scotomata); or hematological complications (thrombocytopenia–platelet count <150,000/μL, disseminated intravascular coagulation, hemolysis); 3. Uteroplacental dysfunction (such as fetal growth restriction, abnormal umbilical artery Doppler waveform analysis, or stillbirth) according to the FIGO guidelines [6]. Pregnant women who met the diagnostic criteria for preeclampsia and with delivery at <34^+0^ weeks of gestation can be subclassified into early-onset preeclampsia. A total of 91 Chinese pregnant women who were diagnosed with early-onset preeclampsia in the Department of Obstetrics and Gynecology of Peking University Third Hospital from January 2010 to May 2021 were included as PE group. Meanwhile, 709 Chinese pregnant women who had normal delivery and single live birth in the department of Obstetrics and Gynecology of Peking University Third Hospital during the same period were selected as the control group (CON). The retrospective study protocol was approved by the Peking University Third Hospital Medical Science Research Ethics Committee (IRB00006761-M2021032).

### 2.2. Clinical and Biochemical Data Collection

Clinical characteristics of patients, such as age of admission, gestational age, disease history, pregnancy history, and blood pressure (1 mmHg = 0.133 kPa), were obtained from electronic medical records. There are 12 risk factors [15] for preeclampsia, which include diabetes, thrombotic diseases, systemic lupus erythematosus (SLE), antiphospholipid syndrome (APS) and kidney diseases, assisted reproductive technology (ART), obstructive sleep apnea, pre-pregnancy body mass index (BMI) > 30 kg/m^2^, age > 35 years old, multiple pregnancies, primipara, and history of eclampsia or preeclampsia. Routine laboratory indicators including albumin (ALB), alanine transaminase (ALT), aspartate transaminase (AST), alkaline phosphatase (ALP), complement C1q, calcium (Ca), creatinine (Cr), C-reactive protein (CRP), cystatin C, γ-glutamyl transpeptidase (GGT), globulin (GLB), triglyceride (TG), total cholesterol (TC), high-density lipoprotein cholesterol (HDL-C), low-density lipoprotein cholesterol (LDL-C), lipoprotein (a) [Lp (a)], apolipoprotein A_1_ (ApoA_1_), apolipoprotein B (ApoB), small dense low density lipoprotein (sdLDL-C), total protein (TP), total bile acid (TBA), total bilirubin (T-Bil), direct bilirubin (D-Bil), uric acid (UA), Urea (UREA), phosphorus (P), absolute value of lymphocyte (LYM), absolute value of neutrophil (NEU), platelet count (PLT), NEU /LTM ratio, PLT /LYM ratio, prothrombin time (PT), prothrombin activity (PTA), activated partial thrombin time (APTT), fibrinogen (FIB), D-Dimer, fibrinogen degradation Products (FDP), thrombin time (TT).

### 2.3. Instruments and Reagents

Fasting blood samples of the participants were collected from elbow venous using vacutainer containing separation glue at 6–10 weeks of gestation. The blood samples were centrifuged at 2793× *g* for 5 min. The serum was separated and stored at −80 °C refrigerator for subsequent detection. Serum liver and kidney function, lipid metabolism, and complement indexes were detected by AU5800 automatic biochemical analyzer (Beckman Coulter, Brea, CA, USA).

The peripheral blood samples were obtained with venipuncture and collected into vacuum blood collection tubes containing sodium citrate as the anticoagulant (INSEPACK^®^ Sekisui, Beijing, China). The plasma was obtained by centrifuging the samples at 1500× *g* for 5 min. Automatic coagulation analyzer (ACL-TOP 700^®,^ Werfen, Barcelona, Spain) was used to detect coagulation items.

The peripheral blood samples were obtained with venipuncture and collected in vacuum blood collection tubes containing EDTA-K2 as the anticoagulant (INSEPACK^®^, Sekisui, Beijing, China). The peripheral leukocytes were counted and classified into neutrophils, eosinophils, basophils, lymphocytes, and monocytes in the traditional five subtype classification method with an automatic blood count analyzer (SYSMEX XN-2000 Automated Hematology Analyzer, Kobe, Japan).

Instrument calibration, calibration, quality control were matched and applied in strict accordance with the standard operation procedure.

### 2.4. Statistical Analysis

SPSS 24.0 and MATLAB software (R2022a) were used for data analysis. The K-S normal distribution was used to detect the normality of data; measurement data conformed to normal distribution with x ± s description and non-normal distribution with a median (interquartile range). Mann–Whitney U test was used for pairwise comparison of skewed distribution data between groups. The count data were tested with chi-square test, and the number of use cases (percentage) was described. *p* < 0.05 was considered statistically significant. 

#### 2.4.1. Logistic Regression Model

All routine laboratory indicators were analyzed with univariate binary Logistic regression; multivariate binary Logistic regression analysis was performed for the variable of *p* < 0.05. The maximum Youden index was taken as the cut-off point, the risk degree was expressed as the OR value [95% confidence interval (CI), 95%CI], and the receiver operating characteristic (ROC) curve was made.

#### 2.4.2. Machine Learning

We used 2 machine learning algorithms: the decision tree model and support vector machine (SVM). For the development of machine learning models, we obtained 12 risk factors and 38 routine laboratory indicators mentioned above. The predictive value of individual risk factors and the models combining risk factors and laboratory indicators were explored, respectively. Machine learning models were trained with all variables as inputs to classify patients likely to have favorable outcomes. Among the study population, 80% were randomly selected for the training set, and the remaining 20% were used as the test set to prevent overfitting of the models. MATLAB version R 2022a was used to train the machine learning models.

##### Decision Tree Model

Decision tree is a commonly used supervised learning algorithm. It uses Gini coefficient, entropy, and other parameters to select features and generate a tree structure, and classifies the original data set into a series of smaller subgroups. This method had the advantages of strong interpretability, low computational costs, and strong robustness. Similarly, the ROC curve was made compared with other models.

##### Support Vector Machine (SVM)

The support vector machine is a learning system that uses a hypothesis space of linear functions in a high-dimensional feature space. This method maximizes the separation boundary of the two classes under the assumption of improving the generalization ability of the classifier. It makes all samples of different classes well discriminated by finding a projection direction and obtaining the optimal hyperplane. In addition, this method can also achieve nonlinear mapping through the kernel function so as to obtain a stronger fitting ability. Among them, the commonly used kernel functions are as follows: gaussian kernel function, polynomial kernel function, sigmoid kernel function, etc. In this study, considering the strong linear relationship between laboratory indicators and predicted results and the objective situation due to limited sample size, a linear kernel with lower complexity was used. We used the ten-fold cross-validation method to verify the ability of the model, and the results of the sensitivity, specificity, and other indicators were good and consistent, which proved that the model had good fitting and generalization ability.

## 3. Results

### 3.1. Participants’ Clinical Characteristics

There were significant differences in maternal age and pre-pregnancy BMI between the two groups (*p* < 0.05), and the PE group had a higher pre-pregnancy BMI compared with the control group (Table 1).

### 3.2. Comparison of Risk Factors 

There was no significant difference in the proportion of pregnant women with thrombotic disease or systemic lupus erythematosus (SLE) between the two groups (*p* > 0.05). However, there were statistically significant differences in the rates of diabetes, antiphospholipid syndrome (APS), kidney disease, obstructive sleep apnea (OSAHS), primipara, history of preeclampsia, and assisted reproductive technology (ART) (*p* < 0.05). The proportion of thrombotic diseases in the PE group was lower than that in the control group, and the other proportions were higher than that in the control group (Table 1).

### 3.3. Comparison of Routine Laboratory Indicators

Among the 38 routine laboratory indicators, there were no significant differences in the levels of PLT/LYM, NEU/LYM, TT, D-Dimer, FDP, TBA, ALP, TP, ALB, GLB, UREA, Cr, P, Cystatin C, HDL-C, Apo-A_1_, and Lp(a) between the two groups (*p* > 0.05). The levels of the rest indicators were all statistically different (*p* < 0.05) (Table 2).

### 3.4. Results of Each Model and Receiver Operating Curve (ROC) Analysis

- Logistic regression analysis

When the risk factors were analyzed with a univariate logistic regression, the results were shown in Supplementary Table S1 (*p* < 0.05).

If only 12 risk factors of PE were analyzed with a multivariate binary logistic regression and an ROC curve analysis was performed, the maximum Youden index of logistic regression was 0.110, the sensitivity of the model was 12.1%, the specificity was 98.9%, and the AUC^ROC^ = 0.78.

Multivariate binary logistic regression analysis was performed on 12 risk factors of PE and 38 routine laboratory indicators. An ROC curve analysis was performed according to the above methods, the maximum Youden index of logistic regression was 0.701, the sensitivity of the model was 73.6%, the specificity was 96.5%, and the AUC^ROC^ = 0.86.

b. Decision tree model analysis

Using a decision tree learning algorithm, if only 12 risk factors of PE were included in the model, the maximum Youden index of logistic regression was 0.130, the sensitivity of the model was 15.4%, the specificity was 97.6%, and the AUC^ROC^ = 0.74; when 12 risk factors of PE and 38 routine laboratory indicators were included in the model, the maximum Youden index of logistic regression was 0.616, the sensitivity of the model was 64.8%, the specificity was 96.8%, and the AUC^ROC^ = 0.77.

c. Support vector machine (SVM) analysis

Using SVM learning system, if only 12 risk factors of PE were included in the model, the maximum Youden index of logistic regression was 0.055, the sensitivity of model was 6.6%, the specificity of model was 98.9%, and the AUC^ROC^ = 0.66. When 12 risk factors of PE and 38 routine laboratory indicators were included in the model, the maximum Youden index of logistic regression was 0.669, the sensitivity of the model was 67.0%, the specificity was 99.9%, and the AUC^ROC^ = 0.93.

**Figure 1:**
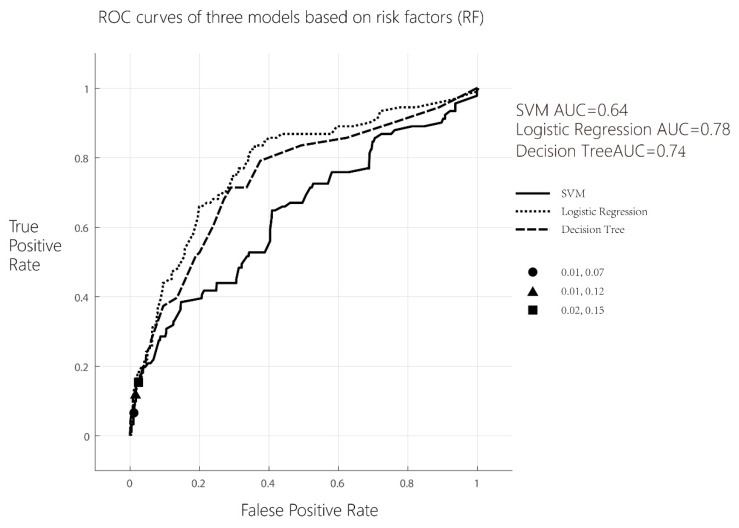
ROC curves of three models based on 12 risk factors (RF).

**Figure 2:**
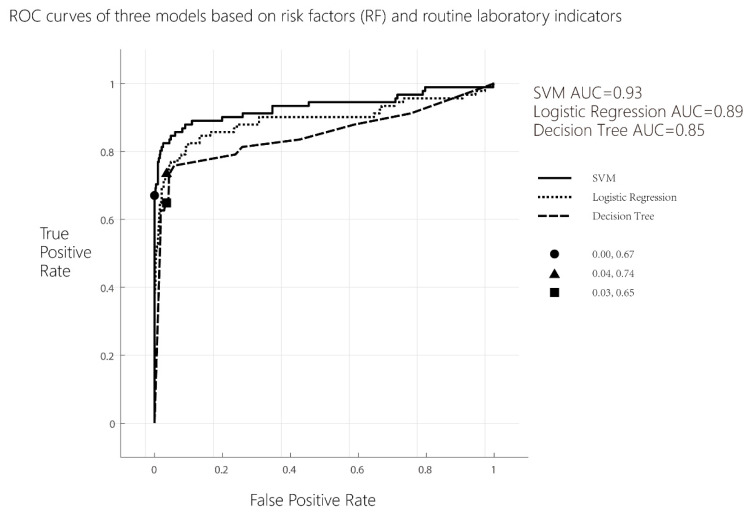
ROC curves of three models based on 12 risk factors (RF) and 38 routine laboratory indicators.

The results of the ROC analysis based on 12 risk factors of PE are shown in Figure 1, and the results of the ROC analysis based on 12 risk factors of PE combining with 38 routine laboratory indicators are shown in Figure 2.

d. Delong test of ROCs differ between models

Delong tests were used to explore whether there were statistical differences in the area under the curve between the three models. If only 12 risk factors of PE were included in the models, the results of the pairwise comparison of ROC curves between support vector machine and decision tree models and those between support vector machine and logistic regression models were statistically different. The results are shown in Table 3 (*p* < 0.05). 

While 12 risk factors of PE and 38 routine laboratory indicators were included in the models, the results of pairwise comparison of ROC curves between support vector machine and decision tree models and that between support vector machine and logistic regression models were also statistically different. The results are shown in Table 4 (*p* < 0.05).

## 4. Discussion

The incidence of PE is related to spiral artery remodeling disorder, endothelial dysfunction, vasospasm, oxidative stress, and micro-embolism. Therefore, factors affecting placenta formation and endothelial function damage are the risk factors for PE [16]. Consistent with other studies [17,18,19], previous history of preeclampsia, diabetes mellitus, thrombotic disease, systemic lupus erythematosus (SLE), antiphospholipid syndrome (APS), kidney disease, assisted reproductive technology, obstructive sleep apnea hypopnea syndrome (OSAHS), BMI > 30 kg/m^2^, age over 35, multiple pregnancies, and primipara were included as risk factors in the model. In this study, there was no thrombotic disease in the PE group, which may be because pregnant women with thrombotic disease tendency continued to take anticoagulant drugs, such as aspirin in the first trimester, effectively preventing abnormal blood flow status and thrombosis and reducing the risk of PE.

The pathologic lesions of preeclampsia and eclampsia are characterized by widespread endothelial lesions in various organ beds [20], such as liver lesions with periportal and portal necrosis and hepatic arterial medial necrosis, based on an autopsy series of 317 mothers who died of eclampsia. Therefore, when PE has not progressed in the first trimester of pregnancy, slight changes in liver vessels may have occurred in pregnant women, and the liver function is affected, which is manifested as elevated liver enzymes, abnormal coagulation function, and abnormal substance metabolism. Similarly, renal tissue demonstrated hallmarks of glomerular endotheliosis reported in previous studies [21]. Glomerular endothelial cell lesions, impaired mechanical barrier and charge barrier, and increased filtration membrane permeability lead to abnormal renal function and proteinuria in PE patients [22].

Previous studies have shown an association between abnormal lipid metabolism and inflammatory activation with preeclampsia [23,24,25,26]. In this study, except for HDL-C and ApoA_1_, the other blood lipid indicators in the PE group were higher than those in the control group. HDL is involved in the reverse transport of cholesterol (as a vascular protective factor that has an anti-atherosclerosis effect while ApoA_1_ is a tool to carry HDL), is also a component of HDL, and has a relatively important role in preventing the occurrence of atherosclerosis. HDL-C and ApoA_1_ levels of the PE group were lower than those of the control group, which was consistent with previous studies [27].

In this study, it is not hard to see that the models established combining routine laboratory indicators with risk factors improve the accuracy of prediction rather than only with risk factors. In this study, the SVM model had the best prediction ability of early-onset PE. Machine learning has received a lot of attention in recent years. The advantages and disadvantages of machine learning and traditional statistical models vary with different research questions, research designs, and research data. Compared to the other machine learning methods, the SVM is very powerful at recognizing subtle patterns in complex datasets, greatly improves the prediction performance of the model, and has a good clinical application prospect [28]. The SVM loss function has its own regular term, so SVM is a structural risk minimization algorithm. The so-called structural risk minimization means to seek a balance between a training error and model complexity to prevent overfitting so as to minimize the real error. In order to better minimize structural risks, regular terms were added to the SVM model construction to further reduce potential overfitting.

In previous studies, mean arterial pressure, uterine arterial pulse index, and serum placental growth factor were selected as biomarkers for early-onset PE prediction [29,30]. Although the accuracy and specificity are relatively high, the collection cost is high, and the operation is difficult. Similar to this study, Jong et al. used logistic regression, decision tree model, naive Bayes classification, support vector machine, random forest algorithm, and stochastic gradient boosting method to build a prediction model for delayed preeclampsia by collecting general clinical data, medical history and biochemical laboratory data. The stochastic gradient boosting model had the best prediction performance with an accuracy and false positive rate of 0.973 and 0.009 [31]. Although different from the variables included in this study, it also shows that machine learning algorithms can effectively predict preeclampsia to a certain extent. The routine laboratory indicators adopted in this study are included in the routine prenatal examination, which is convenient to obtain, simple and rapid, and at the same time reduces the extra cost for patients and the prediction cost. However, the deficiency also lies in this; the established model lacks the specific index of early-onset PE, and the specificity of the model is not high. The sample size of the PE group is smaller than that of the control group, which may have a certain impact on the model. Subsequent studies will improve upon this. The sample size of this study is small, which does not meet the requirements of EPV (Event Per Variable), so the results of the logistic regression may not be robust enough. However, considering that this type of patient is rare and that the results are somewhat interpretable, it is still presented. Further research is needed to confirm the reliability of the results.

## 5. Conclusions

The performance of clinical risk factors alone in predicting early-onset PE is poor, and the performance significantly improved when combing risk factors with routine laboratory indicators. The support vector machine (SVM) model showed the best AUC^ROC^, specificity, and sensitivity compared with the logistic regression model and decision tree model.

## Figures and Tables

**Table 1:** Clinical characteristics and risk factors of Control group and early-onset PE group.

Variables	Control Group (n = 709)	PE Group (n = 91)	Statistical Magnitude	*p* Value
**Age, year**	35 (32–38) ^a^	33 (31–35.5) ^a^	4.32 ^b^	<0.001 *
**Body mass index, BMI**	21.71 (19.93–23.81) ^a^	23.73 (23.12–24.43) ^a^	−4.68 ^b^	<0.001 *
**Medical history, n (%)**				
**Diabetes**	155 (21.86)	29 (31.87)	4.56 ^c^	0.033 *
**Thrombotic disease**	2 (0.28)	0 (0.00)	<0.001 ^c^	0.998
**Systemic lupus erythematosus (SLE)**	5 (0.71)	3 (3.30)	3.17 ^c^	0.075
**Antiphospholipid syndrome (APS)**	20 (2.82)	7 (7.69)	7.31 ^c^	0.026 *
**Kidney disease**	5 (0.71)	6 (6.59)	16.51 ^c^	<0.001 *
**Obstructive sleep apnea hypopnea syndrome (OSAHS)**	0 (0.00)	2 (2.20)	8.05 ^c^	0.005 *
**History of eclampsia or preeclampsia**	4 (0.56)	6 (6.59)	19.12 ^c^	<0.001 *
**History of gestation, n (%)**	591 (83.36)	51 (56.04)	46.69 ^c^	<0.001 *
**Primipara, n (%)**	238 (33.57)	72 (79.12)	74.97 ^c^	<0.001 *
**Fertilization way, n (%)**				
**Assisted reproductive technology (ART)**	119 (16.78)	32 (35.16)	16.61 ^c^	<0.001 *
**Natural conception**	590 (83.22)	59 (64.84)		

^a^ Expressed as the median (interquartile range). ^b^ Rank sum test: Z value ^c^ Chi-square value * *p* values were statistically different, *p* < 0.05.

**Table 2:** Routine laboratory indicators at 6–10 weeks of gestation between Control group and early-onset PE group.

Variable	Control Group (n = 709)	PE Group (n = 91)	Statistical Magnitude	*p* Value
Blood cell count				
PLT, ×10^9^/L	244 (211–283) ^a^	256.5 (226.25–300.5) ^a^	−2.58 ^b^	<0.001 *
LYM, ×10^9^/L	1.79 (1.48–2.1) ^a^	1.98 (1.74–2.40) ^a^	−3.56 ^b^	<0.001 *
NEU, ×10^9^/L	5.24 (4.28–6.55) ^a^	6.25 (5.24–8.35) ^a^	−4.63 ^b^	<0.001 *
PLT/LYM	135.19 (114.35–163.73) ^a^	125.46 (106.26–158.16) ^a^	1.51 ^b^	0.13
NEU/LYM	2.95 (2.29–3.73) ^a^	2.97 (2.42–4.14) ^a^	−0.97 ^b^	0.33
Index of coagulation function				
PT, s	11.3 (11–11.7) ^a^	10.9 (10.5–11.3) ^a^	5.53 ^b^	<0.001 *
PTA, %	91 (88–96) ^a^	97 (92–102) ^a^	−5.15 ^b^	<0.001 *
APTT, s	31.2 (29.3–33.1) ^a^	29.6 (27.7–31.3) ^a^	4.30 ^b^	<0.001 *
TT, s	13.5 (12.9–14) ^a^	13.4 (12.7–14) ^a^	1.06 ^b^	0.29
FIB, g/L	3.27 (2.92–3.66) ^a^	3.51 (3.1–3.98) ^a^	−3.38 ^b^	<0.001 *
D-Dimer, mg/L	0.15 (0.15–0.18) ^a^	0.15 (0.15–0.17) ^a^	1.13 ^b^	0.26
FDP, μg/mL	2.5 (2.5–2.5) ^a^	2.5 (2.5–2.5) ^a^	−0.79 ^b^	0.43
Liver function index				
ALT, U/L	13 (10–18) ^a^	18 (13–27.5) ^a^	−4.67 ^b^	<0.001 *
AST, U/L	16 (14–19) ^a^	19 (15–25) ^a^	−4.70 ^b^	<0.001 *
T-Bil, umol/L	12 (10–14.8) ^a^	10 (8.9–12.6) ^a^	4.71 ^b^	<0.001 *
D-Bil, umol/L	1.3 (1–1.8) ^a^	1.1 (0.6–1.8) ^a^	2.27 ^b^	0.02 *
TBA, umol/L	1.6 (1–2.3) ^a^	1.4 (1–2.35) ^a^	0.54 ^b^	0.59
ALP, U/L	49 (43–58) ^a^	53 (44–64) ^a^	−1.92 ^b^	0.05
TP, g/L	72.4 (70.1–74.7) ^a^	72 (70.9–74.9) ^a^	−0.24 ^b^	0.81
ALB, g/L	43.01 ± 2.42 ^c^	43.04 ± 3.00 ^c^	−0.08 ^d^	0.94
GGT, U/L	14 (11–18) ^c^	17 (14–28) ^c^	−4.86 ^b^	<0.001 *
GLB, g/L	29 (27–31) ^c^	30 (28–32) ^c^	−1.12 ^b^	0.26
Renal function index				
UREA, mmol/L	3.1 (2.7–3.7) ^a^	3.2 (2.8–3.7) ^a^	−1.09 ^b^	0.28
Cr, umol/L	59 (54–63) ^a^	60 (54–65) ^a^	−0.87 ^b^	0.39
Ca, mmol/L	2.3 (2.24–2.35) ^a^	2.33 (2.27–2.4) ^a^	−3.40 ^b^	<0.001 *
P, mmol/L	1.232 ± 0.136 ^c^	1.228 ± 0.138 ^c^	0.21 ^d^	0.83
UA, umol/L	210 (184–239) ^a^	238 (214–279.75) ^a^	−5.57 ^b^	<0.001 *
Cystatin C, mg/L	0.62 (0.57–0.68) ^a^	0.59 (0.54–0.66) ^a^	1.18 ^b^	0.24
Blood lipid indicators				
TCHO, mmol/L	4.01 (3.56–4.48) ^a^	4.27 (3.87–4.79) ^a^	−3.09 ^b^	<0.001 *
TG, mmol/L	1.06 (0.8–1.43) ^a^	1.39 (1.15–1.83) ^a^	−5.00 ^b^	<0.001 *
HDL-C, mmol/L	1.43 (1.24–1.63) ^a^	1.33 (1.14–1.58) ^a^	1.40 ^b^	0.16
LDL-C, mmol/L	2.16 (1.79–2.53) ^a^	2.38 (2.11–2.93) ^a^	−3.84 ^b^	<0.001 *
ApoA_1_, g/L	1578 (1355–1893) ^a^	1508 (1293–2057) ^a^	0.10 ^b^	0.92
ApoB, g/L	621 (519–738) ^a^	702 (520–828) ^a^	−2.35 ^b^	0.02 *
Lp(a), mg/L	87 (44–188) ^a^	87 (42–188.5) ^a^	0.29 ^b^	0.77
sdLDL-C, mmol/L	0.7 (0.56–0.87) ^a^	0.93 (0.77–1.00) ^a^	−3.33 ^b^	<0.001 *
Complement/inflammatory markers				
CRP, mg/dL	1.04 (0.5–2.6) ^a^	1.71 (0.895–3.68) ^a^	−2.68 ^b^	0.007 *
C1q, mg/L	184 (166–211) ^a^	194 (171.75–224.75) ^a^	−2.11 ^b^	0.03 *

^a^ Expressed as the median (interquartile range). ^b^ Rank sum test: Z value; ^c^ Expressed as mean ± standard deviation (SD); ^d^ Student’s *t*-test: *t*-value; * *p*-values were statistically different, *p* < 0.05.

**Table 3:** Delong test of ROCs differ between models (12 risk factors). Pairwise comparison of ROC curves.

**SVM~Decision Tree**	
Difference between areas	0.08
Standard Error ^a^	0.044
95% Confidence Interval	0.047 to 0.220
z statistic	3.02
Significance level	*p* = 0.0025
**Logistic Regression~Decision Tree**	
Difference between areas	0.04
Standard Error ^a^	0.025
95% Confidence Interval	−0.009 to 0.088
z statistic	1.57
Significance level	*p* = 0.1162
**Logistic Regression~SVM**	
Difference between areas	0.12
Standard Error ^a^	0.034
95% Confidence Interval	0.106 to 0.238
z statistic	5.08
Significance level	*p* < 0.0001

**Table 4:** Delong test of ROCs differ between models (12 risk factors and 38 routine laboratory indicators).

**SVM~Decision Tree**	
Difference between areas	0.07
Standard Error ^a^	0.027
95% Confidence Interval	0.026 to 0.132
z statistic	2.92
Significance level	*p* = 0.0035
**Logistic Regression~Decision Tree**	
Difference between areas	0.09
Standard Error ^a^	0.033
95% Confidence Interval	−0.050 to 0.077
z statistic	0.41
Significance level	*p* = 0.6837
**Logistic Regression~SVM**	
Difference between areas	0.07
Standard Error ^a^	0.023
95% Confidence Interval	0.020 to 0.112
z statistic	2.82
Significance level	*p* = 0.0049

## Data Availability

The data used to support the findings of this study are available from the corresponding author upon request.

## Supplementary Materials

The following supporting information can be downloaded at: https://www.mdpi.com/article/10.3390/life13081648/s1, The univariate logistic regression analysis of risk factors for PE was shown in Supplementary Table S1 (*p* < 0.05).

## Abbreviations

PE: preeclampsia; ML, machine learning; BMI, body mass index; SLE, systemic lupus erythematosus; APS, antiphospholipid syndrome; ART, assisted reproductive technology; OSAHS, obstructive sleep apnea; ALB, albumin; ALT, alanine transaminase; AST, aspartate transaminase; ALP, alkaline phosphatase; C1q, complement C1q; Ca, calcium; Cr, creatinine; CRP, C-reactive protein; GGT, γ-glutamyl transpeptidase; GLB, globulin; TG, triglyceride; TC, total cholesterol; HDL-C, high-density lipoprotein cholesterol; LDL-C, low-density lipoprotein cholesterol; Lp (a), lipoprotein (a); ApoA_1_, apolipoprotein A1; ApoB, apolipoprotein B; sdLDL-C, small dense low density lipoprotein; TP, total protein; TBA, total bile acid; T-Bil, total bilirubin; D-Bil, direct bilirubin; UA, uric acid; UREA, urea; P, phosphorus; LYM, absolute value of lymphocyte; NEU, absolute value of neutrophil; PLT, platelet count; PT, prothrombin time; PTA, prothrombin activity; APTT, activated partial thrombin time; FIB, fibrinogen; FDP, fibrinogen degradation Products; TT, thrombin time; SVM, support vector machine, CI, confidence interval.
