# Supplementary material for: Risk Prediction Model of Early-Onset Preeclampsia Based on Risk Factors and Routine Laboratory Indicators
Source: Life (Basel). 2023 Jul 28;13(8):1648. doi: 10.3390/life13081648 (PMC10455518; doi:10.3390/life13081648)
Supplement: Supplementary file 1 [file life-13-01648-s001.zip › life-2462877-supplementary.pdf]

Supplementary Table S1 Univariate logistic regression analysis of risk factors for PE

Variables	OR	95% CI	P
History of eclampsia or preeclampsia	28.562	5.866-139.086	<0.001 *
Diabetes	1.939	1.104-3.408	0.02 *
Thrombotic disease	0.000	0.000-	0.99
Systemic lupus erythematosus (SLE)	1.189	0.148-9.570	0.87
Antiphospholipid syndrome (APS)	1.057	0.370-3.019	0.92
Kidney disease	9.465	2.044-43.843	0.004 *
Assisted reproductive technology (ART)	1.613	0.907-2.867	0.10
Obstructive sleep apnea hypopnea syndrome (OSAHS)	6346878178.435	0.000-	0.99
Body mass index (BMI)>30	1.116	1.026-1.213	0.01 *
Age>35y	0.455	0.250-0.829	0.01 *
Multiple pregnancy	0.375	0.175-0.801	0.01 *
Primipara	4.472	0.648-30.852	0.13

* P values were statistically different, P<0.05
